# Viral clearance and escape during therapy of COVID-19 outpatients: A prospective cohort study

**DOI:** 10.1016/j.isci.2025.114226

**Published:** 2025-11-26

**Authors:** Guillaume Martin-Blondel, Paul Burgat, Valentin Leducq, Françoise Porrot, Andrea Cottignies-Calamarte, Alejandro De Cruz, Céline Dorival, Raphaelle Romieu-Mourez, Camille Chaubet, Vincent Cazaentre, Xavier Boumaza, Quentin Richier, Benjamin Gaborit, Francois Coustilleres, Vincent Dubée, Florence Ader, Youri Yordanov, Olivier Schwartz, Anne-Geneviève Marcelin, Clovis Lusivika-Nzinga, Fabrice Carrat, Cathia Soulié, Roland Liblau, Timothée Bruel, for the ANRS 0003S CoCoPrev Study Group

**Affiliations:** 1 Department of Infectious and Tropical Diseases, Toulouse University Hospital, 31059 Toulouse, France; 2 Toulouse Institute for Infectious and Inflammatory Diseases, INSERM UMR1291 - CNRS UMR5051 - Toulouse III University, 31024 Toulouse, France; 3 NEO-I3D Research Group, Toulouse University Hospital, 31059 Toulouse, France; 4 Sorbonne Université, Inserm, Institut Pierre Louis d’Epidémiologie et Santé Publique (IPLESP), Paris, France; 5 Assistance Publique-Hôpitaux de Paris (AP-HP), Hôpital Pitié-Salpêtrière, Service de Virologie, Paris, France; 6 Virus and Immunity Unit, Institut Pasteur, Université Paris Cité, Paris, France; 7 Antiviral Activities of Antibodies Group, Institut Pasteur, Université Paris Cité, Paris, France; 8 Hôpital Saint-Antoine, Service de Maladies Infectieuses et Tropicales, Paris, France; 9 Department of Infectious Diseases, University Hospital of Nantes and Centre d'Investigation Clinique 1413, INSERM, Nantes 1064, France; 10 Nantes Université, CHU Nantes, INSERM, Center for Research in Transplantation and Translational Immunology, UMR 1064, Nantes, France; 11 Department of Infectious and Tropical Diseases, Tours University Hospital, Tours 37044 cedex9, France; 12 Service des Maladies Infectieuses et Tropicales, CHU d'Angers, Angers, France; 13 Département des Maladies Infectieuses et Tropicales, Hospices Civils de Lyon, 69004 Lyon, France; 14 Centre International de Recherche en Infectiologie (CIRI), Inserm 1111, Université Claude Bernard Lyon 1, CNRS, UMR5308, École Normale Supérieure de Lyon, University Lyon, Lyon 69007, France; 15 HCL, Hôpital Edouard Herriot, Service d'Accueil Des Urgences, Samu 69, Lyon, France; 16 Vaccine Research Institute, Créteil, France; 17 Département de Santé Publique, Hôpital Saint-Antoine, AP-HP Paris, Paris, France; 18 Department of Immunology, Toulouse University Hospital, Toulouse, France

**Keywords:** Immunology, Virology, public health

## Abstract

The efficacy of neutralizing SARS-CoV-2 monoclonal antibodies (mAbs) in preventing severe COVID-19 has been hindered by the diversity of viral strains and the complexity of patient populations. In this prospective cohort study, we used regression analyses to identify virological, immunological, and clinical factors associated with viral clearance and emergence of escape mutations. We included 114 mainly immunocompromised high-risk patients with mild-to-moderate COVID-19 who received mAbs or direct antivirals to prevent COVID-19 progression. Nasopharyngeal SARS-CoV-2 RNA level at day 7 was independently associated with viral load at day 0, serum neutralization at day 7, and treatment received. The emergence of mutations within Spike was observed in 21.9% of patients, all being immunocompromised treated by Sotrovimab or Tixagevimab/Cilgavimab after Omicron emergence, and was independently associated with higher viral load and serum neutralization at day 7. Our data show that suboptimal neutralizing antibodies should be avoided in immunocompromised individuals, given the risk of emergence of viral escape mutations.

## Highlights

• Baseline viral load, neutralization, and therapy govern viral clearance
• Poor viral clearance and low neutralization drive antibody-escape mutant emergence
• Spike mutations emerge in immunocompromised patients treated with suboptimal antibodies
• In immunocompromised patients, nirmatrelvir should be used instead of suboptimal antibodies

## Graphical abstract

**Figure:**
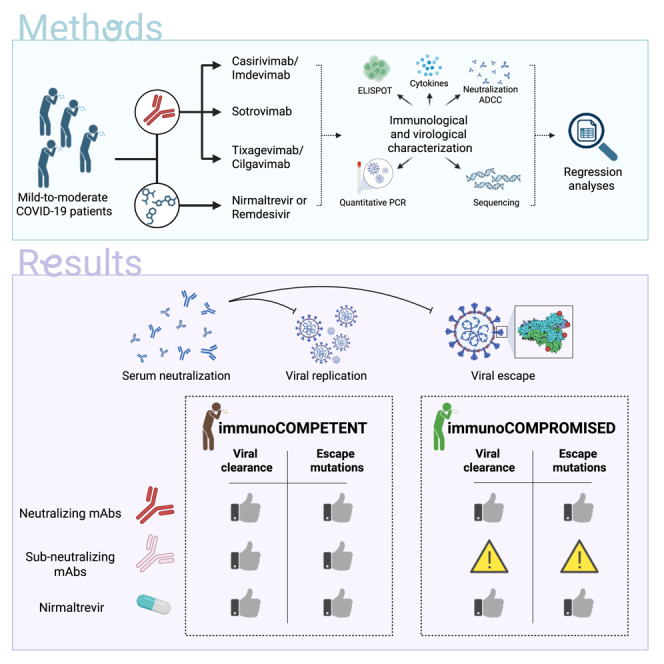

## Introduction

The enduring threat of SARS-CoV-2 infection persists among immunocompromised patients. They demonstrate the highest susceptibility to severe COVID-19 and often necessitate hospitalization with significant mortality.^1^^,^^2^ Their compromised antiviral immune responses enable prolonged detectable SARS-CoV-2 RNA and an extended period of culturable SARS-CoV-2 in respiratory samples.^3^ More importantly, viral replication in the context of an abated antiviral immune response promotes the selection of immune-escape mutations and possibly their accumulation.^4^^,^^5^^,^^6^ This, associated with the exacerbated risk of transmission (because of extended viral shedding), favors the emergence of immune-evasive strains. Accordingly, several studies hypothesized that major variants of concern originate from immunocompromised individuals.^7^

Neutralizing anti-Spike monoclonal antibodies (mAbs) prevent severe COVID-19.^8^ However, viral escape mutations challenge their efficacy. Given the growing prevalence of SARS-CoV-2 sequences carrying Spike that evade neutralization, the first generation of COVID-19 mAbs is now obsolete. Furthermore, the recurrent emergence of variants with increased immune-escape potential led to the use of mAbs with suboptimal neutralizing efficacy.^9^^,^^10^ How reduced neutralization impacts the clinical efficacy of mAb and the risk of therapeutic failure due to intra-host emergence of resistant strains is still ill-defined. The selection of resistant variants in antibody-treated patients also raises public health concerns through possible transmission and spread into the general population.^11^^,^^12^^,^^13^

Emerging viruses, such as highly pathogenic avian influenza viruses, threaten human and global health and highlight the need for effective countermeasures.^14^ Monoclonal antibodies are appealing tools to fight these upcoming viral threats, owing to efficient discovery pipelines, high specificity and low toxicity profiles, and their ability to enhance immune responses.^15^ However, as demonstrated during the COVID-19 crisis, those benefits will face the capacity of viruses to mutate. Therefore, the identification of scenarios where the virus, mAbs, and host’s immune responses favor or prevent the emergence of viral mutations is crucial. Furthermore, an evidence-based framework to guide the use of sub-optimal neutralizing mAb (i.e., against a strain with partial escape) is missing.

To provide insights into these questions, we took advantage of a prospective real-life cohort study of high-risk patients infected with the Delta or Omicron variants of SARS-CoV-2 and treated for mild-to-moderate COVID-19 with either mAb therapies, which were optimal or suboptimal depending on the infecting variant, or with direct antiviral drugs. We performed a comprehensive characterization of patients' immune responses, mAb activities, and viral dynamics. We identified factors associated with the sustained detection of SARS-CoV-2 RNA in respiratory samples and with the emergence of Spike mutations, providing critical insights into the complex interplay between host, virus, and mAb. From these findings, we suggest guidance to optimize the use of therapeutic antibodies against current and future SARS-CoV-2 variants.

## Results

### Characteristics of the patients included

**Figure 1:**
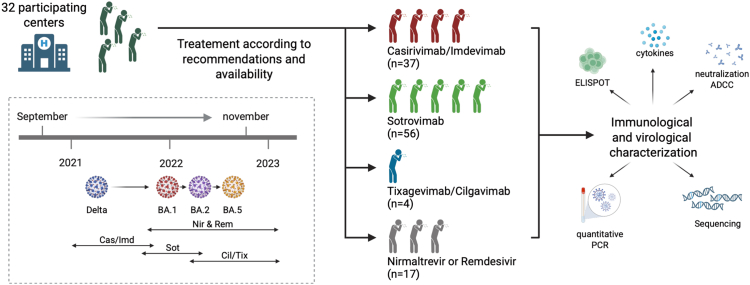
Overview of the study 114 patients at high-risk for severe COVID-19 with PCR-confirmed mild-to-moderate COVID-19 within the five days of symptom onset were administered on day 0 with either mAbs; Casivirimab/Imdevimab (Cas/Imd; *n* = 37), Sotrovimab (Sot; *n* = 56), Tixagevimab/Cilgavimab (Tix/Cil; *n* = 4)) or direct antivirals (*n* = 17, Nirmatrelvir/ritonavir [Nir; *n* = 14] or Remdesivir [Rem; *n* = 3]). We monitored nasopharyngeal SARS-CoV-2 RNA levels, viral sequencing, and blood neutralization, antibody-dependent cellular cytotoxicity (ADCC), T cell responses against N as well as S1 and S2 Spike domains and cytokine/chemokine profiles in samples collected prior to treatment administration (day 0) and at day 7.

**Figure 2:**
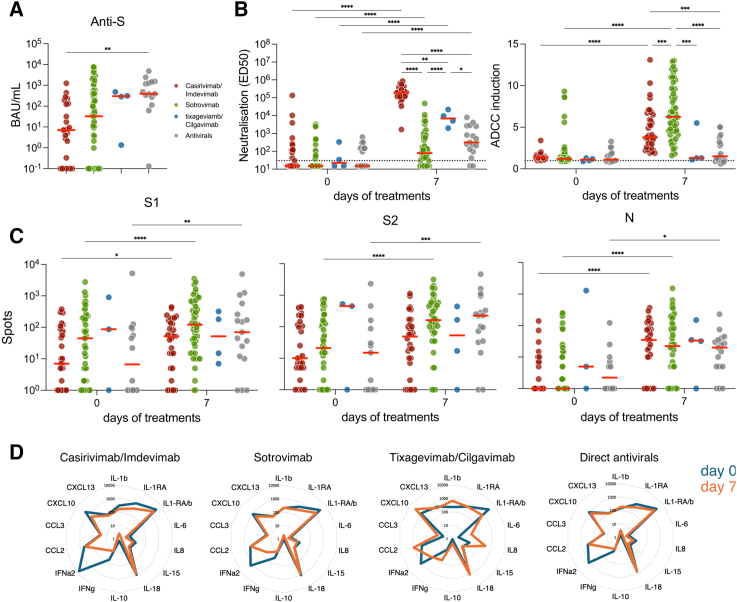
Humoral, cellular and cytokine responses in blood (A) Median titers of anti-S IgG at day 0. Depicted are BAU/mL for each treatment groups. (B) Neutralization (ED_50_: effective dose 50%) and antibody-dependent cellular cytotoxicity (ADCC) induction (fold-change) using the corresponding infecting variant (Delta, BA.1, BA.2 or BA.5) in blood samples obtained at days 0 and 7. (C) T cell responses measured by ELISPOT against S1 and S2 Spike domains and Nucleoprotein at days 0 and 7, expressed as IFNγ spot-forming cells per million of Peripheral blood mononuclear cells (PBMC). (D) Radar plots depicting the median serum cytokines and chemokines profiles measured by electrochemiluminescence-based solid-phase multiplex assay at days 0 and 7. IL-1RA/b indicate the ratio of IL-1RA to IL-1b (IL-1RA/IL-1b). Mixed-model with Tukey’s multiple comparisons post-hoc test; ∗*p* < 0.05; ∗∗*p* < 0.01; ∗∗∗*p* < 0.001; ∗∗∗∗*p* < 0.0001. Each dot represents an individual. Red bars indicate medians. The “Antivirals” group refers to Nirmatrelvir/ritonavir and Remdesivir-treated patients.

Between September 2021 and November 2022, a total of 114 patients were enrolled in the study (Figure 1 and STAR Methods). The characteristics of these patients are depicted in Table 1. The median age was 54.5 years (IQR 44–69), 51% were female, and 77% were immunocompromised. All patients had mild-to-moderate COVID-19, with a median time between symptom onset and initiation of treatment (and inclusion) of 3 days (IQR 2–4). Treatment groups varied by inclusion time, affecting the proportion of immunocompromised patients or those with comorbidities, vaccination status, and the infecting SARS-CoV-2 variant. The type of mAbs or direct antiviral drugs administered was influenced by availability and infecting variant (according to recommendations). All patients infected with the Delta variant received Casirivimab/Imdevimab (*n* = 37), while patients infected with Omicron and its subvariants (*n* = 78) received Sotrovimab (56/78, 71.8%), Tixagevimab/Cilgavimab (4/78, 5.1%), or direct-acting antivirals (17/78, 21.8%; Nirmatrelvir/r [*n* = 14] or Remdesivir [*n* = 3]). The median titer of anti-S IgG at day 0, which was 35.85 BAU/mL (0.05–396.99), differs across and within groups. Individuals treated with Casirivimab/Imdevimab had low levels of anti-S (median of 7 BAU/mL), while those treated with Tixagevimab/Cilgavimab or antivirals had a median of 295 and 389 BAU/mL, respectively. Those treated with Sotrovimab harbor an intermediate profile, with a median of 32 BAU/mL. These differences likely reflect previous SARS-CoV-2 infection or vaccination, because of the evolving vaccine accessibility and recommendations during the study period (Figure 2A; Table 1). Out of the 100 patients for whom results were available, only 4 had detectable anti-N IgG at day 0. One month after inclusion, 4 out of the 106 patients with known outcomes experienced worsening symptoms, including one who was hospitalized for COVID-19.

**Table 1:** Characteristics of the 114 patients included according to treatment

	Overall *N* = 114	Cas/Imd^a^ *N* = 37	Sot^b^ *N* = 56	Nir^c^ *N* = 14	Tix/Cil^d^ *N* = 4	Rem^e^ *N* = 3	*p*-value^f^
Median age (Q1-Q3)	54.5 (44–69)	49 (40–64)	54.5 (44–69)	56.0 (53.0–75.0)	66.5 (51–71)	78.0 (63.0–88.0)	0.099
≥80 years old – N(%)	10 (8.8)	2 (5.4)	5 (8.9)	2 (14.3)	0 (0.0)	1 (33.3)	0.495
Age (min – max)	(23–96)	(29–83)	(23–87)	(40–96)	(38–73)	(63–88)	–
Median BMI (Q1-Q3)	26 (23–31)	29 (23–37)	25 (23–31)	27 (20–30)	21.5 (19–24.5)	24 (24–38)	0.246
Missing	9	7	2	0	0	0	–
Male gender (%)	56 (49.1)	16 (43.2)	28 (50.0)	8 (57.1)	2 (50.0)	2 (66.7)	0.636
**Immunocompromised patients (%)**	88 (77.2)	26 (70.3)	51 (91.1)	6 (42.9)	4 (100.0)	1 (33.3)	**<0.001**
Ongoing chemotherapy	17 (14.9)	5 (13.5)	11 (19.6)	0 (0.0)	1 (25.0)	0 (0.0)	0.210
Solid organ transplantation	22 (19.3)	6 (16.2)	13 (23.2)	0 (0.0)	2 (50.0)	1 (33.3)	0.107
Bone marrow transplantation	5 (4.4)	1 (2.7)	4 (7.1)	0 (0.0)	0 (0.0)	0 (0.0)	0.563
Corticosteroids	9 (7.9)	3 (8.1)	6 (10.7)	0 (0.0)	0 (0.0)	0 (0.0)	0.637
Systemic lupus or vasculitis with immunosuppressive medications	6 (5.3)	1 (2.7)	2 (3.6)	2 (14.3)	1 (25.0)	0 (0.0)	0.253
Immunosuppressive therapy, including RTX	54 (47.4)	16 (43.2)	32 (57.1)	4 (28.6)	2 (50.0)	0 (0.0)	0.132
Cancer	7 (6.1)	0 (0.0)	7 (12.5)	0 (0.0)	0 (0.0)	0 (0.0)	**0.031**
**Comorbidities (%)**	67 (58.8)	23 (62.2)	27 (48.2)	12 (85.7)	2 (50.0)	3 (100.0)	**0.031**
Obesity BMI>30	27 (23.7)	12 (32.4)	10 (17.9)	4 (28.6)	0 (0.0)	1 (33.3)	0.248
COPD and chronic respiratory failure	4 (3.5)	1 (2.7)	3 (5.4)	0 (0.0)	0 (0.0)	0 (0.0)	>0.999
High blood pressure	10 (8.8)	3 (8.1)	3 (5.4)	3 (21.4)	0 (0.0)	1 (33.3)	0.158
Congestive heart failure	7 (6.1)	1 (2.7)	2 (3.6)	3 (21.4)	0 (0.0)	1 (33.3)	**0.037**
Diabetes (type 1 and 2)	12 (10.5)	3 (8.1)	5 (8.9)	3 (21.4)	1 (25.0)	0 (0.0)	0.293
Chronic kidney disease	14 (12.3)	6 (16.2)	5 (8.9)	2 (14.3)	0 (0.0)	1 (33.3)	0.542
Other chronic pathologies	19 (16.7)	4 (10.8)	9 (16.1)	4 (28.6)	2 (50.0)	0 (0.0)	0.317
**Vaccination status (%)**	–	–	–	–	–	–	**<0.001**
Complete (≥3 doses)	76 (69.1)	17 (45.9)	43 (78.2)	12 (100.0)	3 (75.0)	1 (50.0)	–
Incomplete (≤2 doses) or unvaccinated	34 (30.9)	20 (54.1)	12 (21.8)	0 (0.0)	1 (25.0)	1 (50.0)	–
Missing	4	0	1	2	0	1	–
**SARS-CoV-2 variant(%)**	–	–	–	–	–	–	<0.001
Delta	35 (31.0)	35 (97.2)	0 (0.0)	0 (0.0)	0 (0.0)	0 (0.0)	–
Omicron	78 (69.0)	1 (2.8)	56 (100.0)	14 (100.0)	4 (100.0)	3 (100.0)	–
BA.1	28 (35.9)	1 (100.0)	26 (46.4)	1 (7.1)	0 (0.0)	0 (0.0)	–
BA.2	39 (50.0)	0 (0.0)	30 (53.6)	8 (57.1)	1 (25.0)	0 (0.0)	–
Other	11 (14.1)	0 (0.0)	0 (0.0)	5 (35.7)	3 (75.0)	3 (100.0)	–
Missing	1	1	0	0	0	0	–
**Severity of COVID-19 (%)**	–	–	–	–	–	–	0.342
Mild	106 (93.8)	33 (89.2)	53 (96.4)	13 (92.9)	4 (100.0)	3 (100.0)	–
Moderate	7 (6.2)	4 (10.8)	2 (3.6)	1 (7.1)	0 (0.0)	0 (0.0)	–
Missing	1	0	1	0	0	0	–
**Time between symptom onset and initiation of treatment (days, Q1- Q3)**	3.0 (2.0–4.0)	3.0 (2.0–4.0)	3.0 (2.0–4.0)	–	3.0 (1.5–4.0)	3.0 (1.0–3.0)	0.950
Missing	16	1	1	14	0	0	–

a Casirivimab/imdevimab

b Sotrovimab

c Nirmatrelvir/Ritonavir

d Remdesivir

e Tixagevimab/Cilgavimab

f was performed only for the comparisons of Cas/Imd, Sot and Nir groups

### Humoral, cellular, and cytokine levels in blood

In blood samples collected at inclusion (day 0) and day 7, we assessed the neutralizing and antibody-dependent cellular cytotoxicity (ADCC) activities (using infecting viral strains and D614G as control; Figure 2B), measured T cell responses by ELISPOT (using peptide pool from the Omicron or Wuhan strain; Figure 2C), and quantified serum cytokines and chemokines (Figure 2D).

We first performed an unadjusted visualization to describe the dataset. At day 0, all groups displayed low levels of neutralization and ADCC against their infecting variant. At day 7, individuals treated with Casirivimab/Imdevimab displayed the highest level of neutralization, followed by those who received Tixagevimab/Cilgavimab. Patients receiving Sotrovimab demonstrated increased neutralizing activity, but to an extent similar to that of patients treated with direct antivirals (Nirmatrelvir/r or Remdesivir). Levels of ADCC also differed across groups but followed a different pattern. Only patients treated with Casirivimab/Imdevimab and Sotrovimab showed an increase in ADCC capacity at day 7, with Sotrovimab eliciting higher levels.

There were no detectable differences in the T cell responses measured by ELISPOT against S1, S2, and N across treatments (Figure 2C). Anti-N T cells displayed the most consistent induction between day 0 and 7, most likely because of lower baseline levels. There were no detectable differences in the cytokine profiles across treatment groups (Figure 2D). None of the 13 cytokines tested exhibited increased concentrations between day 0 and 7. Rather, IFNα2, IFNγ, and CXCL13 were decreased in 3 out of the 4 groups (Casirivimab/Imdevimab, Sotrovimab, and antivirals; Figures 2D; S1). Although no major difference was detected across treatment groups at day 7, some differences existed at inclusion (Figure S1). The Casirivimab/Imdevimab group displayed higher levels of type I interferon response (as evidenced by IFNα2 and CXCL10) and IL-1β. The Tixagevimab/Cilgavimab group had a tendency for higher CXCL13 levels.

### SARS-CoV-2 viral load in nasopharyngeal swabs

**Figure 3:**
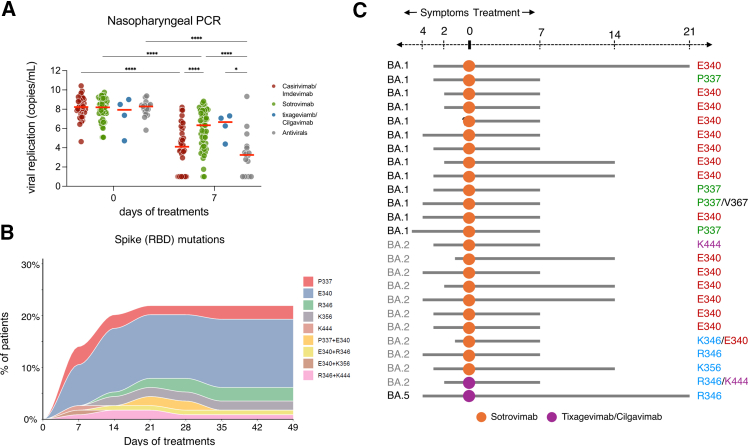
SARS-CoV-2 viral load and spike mutations in nasopharyngeal swabs (A) SARS-CoV-2 RNA levels of the Nucleoprotein gene at days 0 and 7 assessed by RT-PCR. (B) Proportion of the 114 treated patients who experienced the emergence of spike missense mutations in the RBD of Spike, and the type of mutations. Mutations were identified by whole genome sequencing. (C) Schematic representation of the day of emergence (gray bars) and of the Spike missense mutations identified in 25 immunocompromised patients according to the SARS-CoV-2 subvariant and the treatment received. Mixed-model with Tukey’s multiple comparisons post-hoc test; ∗*p* < 0.05; ∗∗*p* < 0.01; ∗∗∗*p* < 0.001; ∗∗∗∗*p* < 0.0001. Each dot represents an individual. Red bars indicate medians. The “Antivirals” group refers to Nirmatrelvir/ritonavir and Remdesivir-treated patients.

Next, we measured SARS-CoV-2 viral shedding longitudinally in NP swabs. An initial unadjusted visualization of the data shows that all treatment groups harbor similar viral loads at baseline, but not at day 7 (Figure 3A). Patients treated with Casirivimab/Imdevimab or direct antivirals displayed a marked reduction of viral replication, up to a 100-fold decrease. Patients receiving Sotrovimab or Tixagevimab/Cilgavimab also exhibited a decrease as compared to day 0, but to a much lower extent (5–10-fold).

Then, we aimed to identify factors associated with the levels of SARS-CoV-2 RNA in NP samples collected at day 7. We used linear regression analysis to identify the factors independently associated with the SARS-CoV-2 viral load in NP swabs at day 7 (Table 2). We excluded the patients who had received Remdesivir or Tixagevimab/Cilgavimab due to the very small sample size of those treatment groups (*n* = 3 and *n* = 4, respectively). All variables with *p*-value less than 0.10 in the bivariate analyses (treatment, age, BMI, gender, immunosuppression status, comorbidities, serum CXCL13 concentration at D0, titer of anti-S at D0, serum neutralization of infecting variant at D0, baseline viral load, serum neutralization of infecting variant at D7 and serum ADCC of infecting variant at D7), as well as clinically relevant variables (vaccine status and IFN-α2a) were included in the multivariate analysis (total of 14 variables; “full model”). By applying the “10 observations per variable” rule, the maximum number of predictors was limited to 11 (“final model”; Table 2). Treatment with Nirmatrelvir/Ritonavir, baseline levels of SARS-CoV-2 viral load, and serum neutralization of the infecting variant at day 7 were independently associated with lower SARS-CoV-2 RNA levels in respiratory samples at day 7 (Table 2). The results of the full model (Table S2) are consistent with those of the final model (Table 2). Assessment of skewness and linearity indicated that all continuous variables included in the model met the linearity assumption. Sensitivity analysis stratified by immune status, age, and vaccination status produced similar results in terms of treatment association with the outcome, supporting the robustness of the main findings (Tables S3–S5). Results on non-immunocompromised, >80-year-old, and unvaccinated/partially vaccinated patients were not presented because of the small size of these groups.

**Table 2:** Linear regression analysis of factors associated with SARS-CoV-2 N gene viral load at day 7 (log10 cp/ml) without the 3 Remdesivir and the 4 Tixagevimab/Cilgavimab treatments (*N* = 107)

	Bivariate analysis Coefficient [95%CI] (*p*-value)	Multivariable analysis Coefficient [95%CI] (*p*-value)
Sotrovimab Casirivimab/Imdevimab Nirmatrelvir/ritonavir	Ref. −2.04 [-3.02; -1.07] (<0.001) −3.27 [-4.65; -1.90] (<0.001)	Ref. −0.01 [-2.02; 2.01] (0.996) −3.18 [-4.69; -1.67] (<0.001)
Age (per 5 years)	0.18 [0.02; 0.33] (0.025)	0.10 [-0.04; 0.24] (0.170)
BMI (kg/m^2^)	−0.09 [-0.16; -0.02] (0.008)	−0.05 [-0.11; 0.01] (0.098)
Gender (male vs. female)	1.20 [0.21; 2.18] (0.018)	0.52 [-0.34; 1.38] (0.232)
Immunocompromised patients (vs. non immunocompromised)	1.61 [0.44; 2.77] (0.008)	0.31 [-0.77; 1.40] (0.567)
Vaccine status incomplete (vs. complete)	−0.21 [-1.31; 0.88] (0.699)	−0.05 [-1.01; 0.92] (0.924)
Serum CXCL13 concentration at D0 (log10 pg/ml)	1.44 [0.07; 2.82] (0.040)	0.92 [-0.29; 2.12] (0.135)
Serum neutralization of the infecting variant at D0 (log10 titer)	−1.23 [-1.86; -0.59] (<0.001)	−0.26 [-0.91; 0.38] (0.418)
SARS-CoV-2 N gene viral load at D0 (log10 cp/ml)	0.75 [0.32; 1.17] (0.001)	0.55 [0.17; 0.93] (0.005)
Serum neutralization of the infecting variant at D7 (log10 titer)	−0.62 [-0.91; -0.34] (<0.001)	−0.63 [-1.21; -0.05] (0.035)
Serum ADCC of the infecting variant at D7 (fold-change)	0.21 [0.05; 0.36] (0.009)	−0.07 [-0.22; 0.08] (0.367)

### SARS-CoV-2 spike mutations in nasopharyngeal swabs

Finally, we investigated the emergence of mutations in the RBD domain of the Spike protein. Among the 114 patients, 25 (21.9%) had emergence of missense mutations at positions P337, E340, R346, K356, and K444, which are all known antibody escape positions (Figure 3B). A large majority of the mutations were concentrated at position E340. The median sequencing depth was 1275 [682–2488] in the Spike protein and 698 [260–1829] in the RBD domain of the Spike protein (Table S7). Emerging mutations were never detected at frequencies below 20% (Figure S2). Among the 25 patients who developed mutations, 15/25 (60%) had a positive viral load after day 7, allowing sequencing, and only 8/25 (32%) after day 14.

For further analyses, we excluded the 2 patients with viral emergence who had received Remdesivir or Tixagevimab/Cilgavimab due to the very small sample size of those treatment groups (*n* = 7). We therefore focused on 23 patients who developed mutations within the Casirivimab/Imdevimab, Sotrovimab, and Nirmatrelvir groups (*n* = 107). All patients harboring mutant RBD were immunocompromised patients (23/23, versus 60/84 [72.3%] of those with non-mutant RBD), infected by Omicron variants (23/23, versus 48/84 [67.6%]), and received either Sotrovimab (*n* = 23/23) (Figure 3C; Table S1). None of the patients who received Casirivimab/Imdevimab (*n* = 37, all infected with the Delta variant), Nirmatrelvir/r (*n* = 14, all infected with Omicron variants) experienced mutations in the SARS-CoV-2 S gene. Patients infected with SARS-CoV-2 harboring missense mutations in the RBD also had higher median SARS-CoV-2 NP viral load than patients without mutations at day 0, 8.5 (8–9.1) log_10_ copies/mL versus 8.1 (7.2–8.8) log_10_ copies/mL (*p* = 0.022), and at day 7, 7.7 (7–8) log_10_ copies/mL versus 4.1 (2.9–5.9) log_10_ copies/mL (*p* < 0.001). Serum cytokines and chemokines concentrations at day 0 and day 7 were globally comparable between the 2 groups, as were SARS-CoV-2-specific T cell responses. However, patients infected with SARS-CoV-2 with mutant RBD in the Spike gene had lower serum neutralization titers on day 7 for both D614G (531.7 (367–915.2) versus 38,549.5 (1,322.5–226,711.5), *p* < 0.001) and their infecting variant (48.2 (15–116.9) versus 4,432 (135.5–179,926.5), *p* < 0.001) at day 7. The serum ADCC titers at day 0 on both D614G (1.1 (1.1–1.3) versus 1.3 (1.2–1.8), *p* = 0.016) and their infecting variant (1.2 (1.1–1.29) versus 1.4 (1.2–1.8), *p* = 0.042) were lower in patients exhibiting SARS-CoV-2 RBD mutations. However, the serum ADCC titers were higher on both the D614G (8.7 (6.1–9.9) versus 4.9 (3.6–6.9), p=<0.001) and the infecting variant 7.3 (4.6–11) versus 4.1 (2.7–6.7), p=<0.001) at day 7 in these patients, a finding that could reflect a overrepresentation of Sotrovimab treatment in this group. Finally, the proportions of patients experiencing clinical worsening at day 7 or day 28, as well as those hospitalized for COVID-19, did not differ between patients who developed missense mutations and those who did not (Table S1). Time to clinical recovery and time to viral clearance were not collected in this study.

To identify the factors independently associated with the emergence of escape mutations in the Spike gene during the follow-up period, we conducted a logistic regression analysis (Table 3). Because only immunocompromised individuals developed these mutations, we restricted the analysis to the 83 immunocompromised patients, again excluding the 7 patients who had received Remdesivir (*n* = 3) or Tixagevimab/Cilgavimab (*n* = 4). The NP viral load at day 7 and serum neutralization of the infecting variant at day 7 were associated with the emergence of mutations in the Spike gene (Table 3). Results remain similar when LASSO selection was used (Table S6).

**Table 3:** Logistic regression analysis of factors associated with emergence of amino acid-substitution mutation in 83 immunocompromised patients (23 patients with emergence vs. 60 patients without emergence with imputing data and without Tixa/Cilga or Remdesivir treatments)

	Bivariate analysis OR [95%CI] (*p*-value)	Multivariable analysis OR [95%CI] (*p*-value)
Age (per 5 years)	1.20 [1.02 – 1.44] (0.038)	0.84 [0.58–1.13] (0.278)
Vaccine status incomplete (vs. complete)	0.89 [0.28–2.57] (0.838)	1.83 [0.25–15.65] (0.553)
Serum IFN-α2a concentration at D0 (log10 pg/ml)	0.98 [0.60–1.63] (0.933)	0.78 [0.30–1.95] (0.599)
Serum neutralization of the infecting variant at D0 (log10 titer)	0.44 [0.12–1.11] (0.142)	–
Serum ADCC titer of the infecting variant at D0 (fold-change)	0.90 [0.57–1.22] (0.573)	–
SARS-CoV-2 N gene viral load at D0 (log10 cp/ml)	1.84 [1.15 – 3.24] (0.020)	2.03 [0.92–5.71] (0.120)
Serum neutralization of the infecting variant at D7 (log10 titer)	0.29 [0.13 – 0.51] (<0.001)	0.38 [0.14 – 0.77] (0.022)
Serum ADCC of the infecting variant at D7 (fold-change)	1.35 [1.15 – 1.63] (<0.001)	1.33 [0.98–1.92] (0.092)
SARS-CoV-2 N gene viral load at D7 (log10 cp/ml)	2.75 [1.79 – 4.90] (<0.001)	2.96 [1.58 – 6.93] (0.003)

## Discussion

In this cohort of 114 patients treated within the first five days of mild-to-moderate COVID-19 associated with different variants, 77% were immunocompromised, we investigated the effects of different treatments on viral load and immune responses. Patients received either combination therapy with mAbs (Casirivimab/Imdevimab), functional suboptimal monotherapy (i.e., only one out of the two mAbs is active; Cilgavimab/Tixagevimab), sub-optimal monotherapy (Sotrovimab), or direct antivirals (Nirmatrelvir/ritonavir or Remdesivir).

The administration of these therapeutic antibodies had a profound impact on serum neutralization and ADCC at day 7. This is expected, but our data demonstrate that the exogenous mAbs add to the endogenous immune response, and may be used to modulate circulating antibody functions even in immunocompetent individuals. In contrast to the humoral compartment, antibody infusion had no detectable impact on T cells responses and cytokine levels, as previously observed.^16^ However, we observed major differences in viral clearance between groups, with all individuals receiving mAbs sub-optimally active against their infecting variant (i.e., Sotrovimab against BA.1 or BA.2 and Cilgavimab/Tixagevimab against BA.2 and BA.5), demonstrating higher NP viral load at day 7.

We further show that the viral load at day 7 is independently associated with the initial viral load, the serum neutralization at day 7, and the treatment received. We do not detect such an association with ADCC or T cell response. This indicates that serum neutralization, whether induced by therapeutic mAbs or endogenous response, or the administration of effective antiviral treatment, is the key factor determining the evolution of the viral load. It is also possible that the small sample size of our study limits our capacity to detect additional (and possibly less strong) correlates of protection. Still, the lack of a detectable association with the ADCC is interesting, as it implies that neutralization surpasses other functions of antibodies to reduce viral load. Our data therefore suggest that it is the low level of neutralization, rather than ADCC, that explains Sotrovimab antiviral efficacy against BA.2 and subsequent variants.^10^^,^^14^^,^^17^ This interpretation is consistent with the observation that low levels of neutralization are sufficient to obtain clinical efficacy with the anti-S antibody Adintrevimab.^18^

Despite antiviral efficacy, a low level of neutralization may drive the selection of escape mutants. Consistently, we observed the emergence of mutations in 21.9% of patients, which was independently associated with higher viral load and lower serum neutralization at day 7. It was also exclusively observed in immunocompromised patients infected with the Omicron variant and treated with suboptimal neutralizing mAbs (i.e., Sotrovimab against BA.1 or BA.2; Cilgavimab/Tixagevimab against BA.2/BA.5). Nasopharyngeal viral load and neutralization were the only parameters independently associated with emergence in our model, unlike another study that identified a cytokine signature using random forest analysis.^16^ Our data suggest that an effective endogenous antibody response may counterbalance a suboptimal therapy. Indeed, it is likely that the level of neutralization required to reduce NP viral load and prevent mutation can be obtained by either a highly effective therapeutic mAb, a strong endogenous antibody production, or a combination of both.

An elevated viral load may be either a cause or a consequence of the emergence of escape mutations. However, given that most patients who develop resistance ultimately clear the resistant virus, it seems more likely that increased viral replication favors the emergence of resistant viruses, rather than the opposite. Consistently, the selection of escape mutants was not associated with any clinical impact in this study. It is well described that highly mutated viruses tend to arise in severely immunocompromised individuals who are unable to clear viral infections, regardless of whether the viruses are wild-type or mutated.^7^ Likewise, our data indicate that resistant viruses do not emerge in immunocompetent patients, suggesting that most intra-host mutations are readily controlled by intact immune responses. Therefore, the emergence of escape mutants is most likely due to a failure in reducing viral replication and primarily poses a risk to treatment efficacy, rather than overall viral clearance.

The optimal management of COVID-19 in immunocompromised patients remains to be defined. Current European^19^^,^^20^ and U.S. guidelines (https://www.idsociety.org/practice-guideline/covid-19-guideline-treatment-and-management/) recommend early treatment of mild-to-moderate COVID-19 immunocompromised patients with Nirmatrelvir/Ritonavir or Remdesivir. In selected patients with profound immunosuppression, combination strategies – such as dual antiviral therapy or antiviral therapy combined with monoclonal antibodies or convalescent plasma—may be considered. Molnupiravir and monoclonal antibodies are considered alternatives, although their use is limited: Molnupiravir because of its lower efficacy and the potential emergence of immune escape variants in immunocompromised hosts,^21^ and monoclonal antibodies, if available. Nirmatrelvir/r and/or Remdesivir could also be contraindicated in some patients, again highlighting the need for alternative strategies. Two antibodies were recently approved to treat COVID-19: Pemivibart and Sipavibart. Sipavibart is discontinued due to its lack of efficacy against strains carrying the Phe456Leu mutation, which is widely prevalent in the recent variant KP3.1.1, XEC, and LP.8.1.^22^^,^^23^^,^^24^ Altogether, we therefore recommend evaluating the combination of Pemivibart with an effective antiviral to treat immunocompromised individuals.

To conclude, our findings emphasize the importance of rapidly controlling viral replication to limit risks of transmission and emergence. The choice of antiviral strategy should consider the patient’s immune status and the potency of the treatment. In immunocompetent individuals considered at high risk of severe COVID-19, the rise of an endogenous antiviral response would allow the use of suboptimal mAbs early on. However, in immunocompromised patients, the use of highly neutralizing mAbs or the combination of suboptimal antibodies with direct antivirals appears crucial. More generally, we recommend always combining antibodies to antivirals to treat immunocompromised individuals when the *in vitro* IC50 of the antibody is reduced by a mutation. This applies to SARS-CoV-2 and its variants but likely also to all respiratory viruses.

### Limitations of the study

While our findings provide valuable insights into the relationship between treatment, immune response, and viral evolution in COVID-19 patients, our study has several limitations. First, the relatively small sample size of 114 patients limits our sensitivity to identify parameters associated with clearance and escape. Heterogeneity in treatment groups and notably in their proportion of immunocompromised patients is also a challenge. Despite the use of multivariate models and sensitivity analyses, we may have missed confounding factors across treatment groups. Further randomized studies are thus needed to confirm our conclusion. Our real-life design also led to baseline differences in anti-S levels across our study groups, as SARS-CoV-2 seroprevalence and COVID-19 vaccination increased during the study period. As the information of a previous infection was not collected directly from participants, the impact of those baseline differences needs to be explored in a subsequent study. Therefore, larger studies that include a precise characterization of the hybrid immunity in each participant may enable the identification of other biomarkers or mechanisms. Additionally, our study did not include a control group that received an optimal antibody matched to the infecting variant, which would have allowed us to directly compare the efficacy of combination therapy versus monotherapy with a highly effective antibody. Furthermore, our study was conducted with a specific set of SARS-CoV-2 variants in real-life settings, making it impossible to perform separate analyses on variants and treatments. It is therefore possible that replicating potentials and immune-evasion properties of viral variants have partly influenced our findings. Finally, we did not comprehensively assess non-neutralizing functions of antibodies, such as antibody-mediated phagocytosis or complement activation, which may also contribute to the antiviral effects of mAbs. Further studies are needed to investigate how non-neutralizing functions beyond ADCC may impact virological and treatment outcomes. Despite these limitations, our study highlights the importance of considering the patient’s immune status and the potency of the mAb when selecting an antiviral strategy.

## Resource availability

### Lead contact

Further information and requests for resources and reagents should be directed to and will be fulfilled by the Lead Contact, Timothée Bruel (timothee.bruel@pasteur.fr).

### Materials availability

All unique/stable reagents generated in this study are available from the corresponding authors with a completed Materials Transfer Agreement.

### Data and code availability

• This study did not generate any new codes.
• The raw sequencing data contains traces of human genomic DNA and cannot be made publicly available according to French law. However, this data is available upon request.
• The viral sequences are available on GISAID

## Acknowledgments

We thank Prof. Yazdan Yazdanpanah and all the ANRS-MIE team for their invaluable support and help. This study would have not been possible without the teams involved in the COCOPREV Study and designated as the COCOPREV Study Group: Magali Garcia, Valentin Giraud, Agathe Metais, France Cazenave-Roblot, Jean-Philippe Martellosio (CHU de Poitiers); Anne-Marie Ronchetti, Thomas Gabas, Naima Hadjadj, Célia Salanoubat, Amélie Chabrol, Pierre Housset, Agathe Pardon, Anne-Laure Faucon, Valérie Caudwell, Latifa Hanafi (CHU Sud Francilien, Corbeil-Essonne); Laurent Alric, Grégory Pugnet, Morgane Mourguet, Eva Bories, Delphine Bonnet, Sandrine Charpentier, Pierre Delobel, Alexa Debard, Colleen Beck, Xavier Boumaza, Stella Rousset (CHU de Toulouse); Fanny Lanternier, Claire Delage, Elisabete Gomes Pires, Morgane Cheminant, Nathalie Chavarot (Hôpital Necker, Paris); Anthony Chauvin, Xavier Eyer; Véronique Delcey (Hôpital Lariboisière, Paris); Simon Bessis, Romain Gueneau (Hôpital du Kremlin Bicêtre); Pelagie Thibaut, Marine Nadal, Martin Siguier, Marwa Bachir, Christia Palacios (Hôpital Tenon, Paris); Valérie Pourcher, Antoine Faycal, Vincent Berot, Cécile Brin, Siham Djebara, Karen Zafilaza, Stephane Marot, Sophie Sayon, Valentin Leducq (Hôpital de la Pitié Salpétrière,Paris); Karine Lacombe, Yasmine Abi Aad, Thibault Chiarabini, Raynald Feliho, Nadia Valin, Fabien Brigant, Julien Boize, Pierre-Clément Thiébaud, Marie Moreau, Charlotte Billard (Hôpital St Antoine, Paris), Nathalie De Castro, Geoffroy Liégeon, Blandine Denis, Jean-Michel Molina, Lucia Etheve (Hôpital Saint Louis, Paris); André Cabié, Sylvie Abel, Ornella Cabras, Karine Guitteaud, Sandrine Pierre-François (CHU de Martinique); Vincent Dubee, Diama Ndiaye, Jonathan Pehlivan, Michael Phelippeau, Rafael Mahieu (CHU d’Angers); Alexandre Duvignaud, Thierry Piston, Arnaud Desclaux, Didier Neau, Charles Cazanave (CHU de Bordeaux); Jean-François Faucher, Benjamin Festou, Magali Dupuy-Grasset, Véronique Loustaud-Ratti, Delphine Chainier (CHU de Limoges); Nathan Peiffer-Smadja, Olivia Da Conceicao, Michael Thy, Lio Collas, Cindy Godard, Donia Bouzid, Vittiaroat Ing, Laurent Pereira, Thomas Pavlowsky, Camille Ravaut (Hôpital Bichat, Paris); Antoine Asquier-Khati, David Boutoille, Marie Chauveau, Colin Deschanvres, François Raffi (CHU de Nantes); Audrey Le Bot, Marine Cailleaux, François Benezit, Anne Maillard, Benoit Hue, Pierre Tattevin (CHU de Rennes); François Coustilleres, Claudia Carvalho-Schneider, Simon Jamard, Laetitia Petit, Karl Stefic (CHU de Tours); Natacha Mrozek, Clement Theis, Magali Vidal, Leo Sauvat, Delphine Martineau (CHU de Clermond-Ferrand); Benjamin Lefèvre, Guillaume Baronnet, Agnès Didier (CHRU de Nancy); Florence Ader, Thomas Perpoint, Anne Conrad, Paul Chabert, Pierre Chauvelot (CHU de Lyon); Aurélie Martin, Paul Loubet, Julien Mazet, Romaric Larcher, Didier Laureillard (CHU de Nîmes); Mathilde Devaux (Hôpital de Poissy); Jérôme Frey, Amos Woerlen, Aline Remillon, Laure Absensur-Vuillaume, Pauline Bouquet (CHU de Metz); Albert Trinh-Duc, Patrick Rispal (Hôpital d’Agen); Philippe Petua, Julien Carillo (Hôpital de Tarbes); Aurore Perrot, Karen Delavigne, Pierre Cougoul, Jérémie Dion, Odile Rauzy (Oncopole, Toulouse) Yazdan Yazdanpanah, Ventzislava Petrov-Sanchez, Alpha Diallo, Soizic Le Mestre, Guillaume Le Meut (ANRS-MIE); Isabelle Goderel, Frédéric Chau, Brahim Soltana, Jessica Chane Tang (IPLESP), Jeremie Guedj (Université de Paris, IAME, INSERM, Paris), Yvanie Caille (Renaloo).

The ANRS0003S COCOPREV cohort is conducted with the support of 10.13039/501100003323ANRS│10.13039/100024438MIE and funded by French ministries: 10.13039/501100014630Ministère des Solidarités et de la Santé and 10.13039/100012948Ministère de l'Enseignement supérieur, de la Recherche et de l'Innovation». T.B. was supported by the 10.13039/501100001665French National Agency for Research (ANR-23-CE15-0039-01).

## Author contributions

Conceptualization: G.M.B., Y.Y., A.G.M., F.C., C.S., R.L., and T.B. Formal analysis: G.M.B., P.B., V.L., A.C.C., A.G.M., C.L.N., F.C., C.S., R.L., and T.B., investigation: V.L., F.P., A.C.C., A.D.C., K.L., F.R., F.C., V.D., F.A., R.M.M., C.C., T.B.; resources: G.M.B., D.C., Y.Y., and A.G.M. Writing - original draft: G.M.B. and T.B. Writing - review and editing: G.M.B., P.B., V.L., F.P., A.C.C., A.D.C., K.L., F.R., F.C., V.D., F.A., C.D., R.M.M., C.C., Y.Y., O.S., A.G.M., C.L.N., F.C., C.S., R.L., and T.B. Visualization: G.M.B., P.B., V.L., C.L.N., and T.B. Supervision: G.M.B., O.S., A.G.M., F.C., C.S., R.L., and T.B. Funding acquisition: G.M.B., O.S., A.G.M., F.C., C.S., R.L., and T.B.

## Declaration of interests

T.B. has served as a speaker for AstraZeneca. Other authors and investigators have no conflict of interest to disclose.

## STAR★Methods

### Key resources table

**Table:** 

REAGENT or RESOURCE	SOURCE	IDENTIFIER
**Antibodies**		

Sotrovimab	Kind gift of Dr Thierry Prazuck (CHR d’Orléans, France)	N/A
anti-IgG Alexa Fluor 647	Jackson ImmunoResearch	Cat#A-21445

**Virus Strains**		

D614G(hCoV-19/France/GE1973/2020)	National Reference Center for Respiratory Viruses (Institut Pasteur, Paris, France)	EPI_ISL_41463
Delta	Laboratory of Virology of Hopital Européen Georges Pompidou (Assistance Publique – Hopitaux de Paris)	EPI_ISL_2029113
Omicron BA.1	NRC UZ/KU Leuven, Belgium	EPI_ISL_6794907
Omicron BA.4	NRC UZ/KU Leuven, Belgium	EPI_ISL_15728568
Omicron BA.5	CHU de Tours, France	EPI_ISL_13660702

**Chemicals, Peptides, and Recombinant Proteins**		

Hoechst 33342	Invitrogen	Cat#H3570
Paraformaldehyde 4%	Alfa Aesar	Cat#J19943.K2
Spike peptide pool	JPT-Peptide-Technologies	PM-SARS2-SMUT08-2
Nucleoprotein peptide pool	JPT-Peptide-Technologies	PM-WCPV-NCAP-2
CEF peptide pool	Cellular Technology	CTL-CEF-001

**Critical commercial assays**		

ADCC Reporter Bioassay	Promega	Cat#G7010
Bright-Glo Luciferase Assay System	Promega	Cat#E2620
U-PLEX Biomarker Group 1 kit	Meso Scale Discovery	Custom assay
S-PLEX assay #1	Meso Scale Discovery	Custom assay
S-PLEX assay #2	Meso Scale Discovery	Custom assay
R-PLEX assay	Meso Scale Discovery	Custom assay
IFNγ ELISPOT kit	Medix Biochemica Diaclone	856.051
TaqPath™ COVID-19 RT-PCR assay	ThermoFisher	Cat# A51738
xGen™ ARTIC nCoV-2019 Amplicon Panel v4.1	Integrated DNA Technologies	Cat#10011442

**Experimental Models: Cell lines**		

293T	ATCC	Cat#CRL-3216
U2OS cells	ATCC	Cat#HTB-96

**Software and Algorithms**		

Harmony High-Content Imaging and Analysis Software	PerkinElmer	Cat#HH17000012
Excel 365	Microsoft	https://www.microsoft.com/en-ca/microsoft-365/excel
Prism 8	Graphpad	https://www.graphpad.com/
FlowJo v10	Tree Star	https://www.flowjo.com/
Discovery Workbench® 4.0	Meso Scale Discovery	https://www.mesoscale.com/
MinKNOW	Oxford Nanopore Technologies	https://nanoporetech.com/document/experiment-companion-minknow
ARTIC SARS-CoV-2 Workflow (v0.3.12)	EPI2ME labs	https://github.com/epi2me-labs/wf-artic
Nextclade	Nextstrain	https://nextstrain.org/
Pangolin	© SARS-CoV-2 lineages	https://cov-lineages.org/
PEPPER-Margin-DeepVariant pipeline (v0.8)	Open Access	https://github.com/kishwarshafin/pepper
R v4.4.1.	Comprehensive R Archive Network (CRAN)	https://cran.r-project.org/

**Deposited Data**		

Viral genome sequences	GISAID	EPI_ISL_41463 EPI_ISL_2029113 EPI_ISL_6794907 EPI_ISL_15728568 EPI_ISL_13660702

### Experimental models and subject details

#### Human subjects

Our study is an ancillary investigation of the “Agence Nationale de Recherche sur le SIDA et les hépatites virales” (ANRS) “Prévention des complications de la COVID-19” (CoCoPrev) study (NCT04885452), a multicentric prospective cohort study encompassing patients at high risk for severe COVID-19.^25^ This cohort was set-up in September 2021 to evaluate clinical and virological outcomes of patients with mild-to-moderate COVID-19 treated under an emergency use authorization (EUA). Patients were eligible if they exhibited PCR-confirmed mild-to-moderate COVID-19 within five days of symptom onset and received treatment. Treatment was determined at the discretion of the treating physician. During the study period, patients received either a single infusion of Casirivimab/Imdevimab,^26^ of Sotrovimab,^27^ of Tixagevimab/Cilgavimab,^28^ or 5 days of oral Nirmatrelvir/Ritonavir,^29^ or 3 days of intravenous Remdesivir.^30^ Baseline demographics, clinical and biological data were collected during the inclusion visit (day 0, prior to treatment initiation), then at day 7 and month 1. Nasopharyngeal (NP) swabs were collected at days 0 and 7, and repeated weekly in patients with a SARS-CoV-2 PCR with a cycle threshold (CT) < 31. Blood samples were obtained at days 0 and 7. The protocol has been approved by the “CPP Sud-Est IV” Ethics Committee (Paris, France) and the French Regulatory Authority. Written informed consent was obtained from each patient. The primary outcome was the SARS-CoV-2 RT-PCR viral load in NP swabs. The secondary outcome was the emergence of any missense mutation in the Spike receptor binding domain (RBD) with frequency >2%.^31^^,^^32^^,^^33^ All biological samples were centralized and stored at “Center de Ressources Biologiques ANRS-MIE”, Bordeaux, France.

#### PBMCs cryopreservation

PBMCs were obtained from EDTA tubes within 4 h after the venipuncture. Isolation was performed by density-gradient sedimentation using Ficoll-Paque (Pancoll, Pan-Biotech). PBMCs were frozen in heat-inactivated fetal bovine serum (Gibco FBS, ThermoFisher) containing 10% tissue culture-grade DMSO (Sigma). Cryovials containing 10-15.10^6^ cells were transferred at −80°C at a cooling rate of 0.2°C–1 °C/min using a cryopreservation module and transferred to liquid nitrogen after 24 to 96 h until use.

#### Viral strains

All strain were previously described.^34^^,^^35^^,^^36^^,^^37^ The sequences of the isolates were deposited on GISAID immediately after their generation, with the following ID: EPI_ISL_41463 (D614G); EPI_ISL_2029113 (Delta); EPI_ISL_6794907 (Omicron BA.1); EPI_ISL_15728568 (Omicron BA.2); EPI_ISL_13660702 (Omicron BA.5). Viral stocks were produced on Vero E6 or IGROV-1 cells and titrated in limiting dilution on Vero E6 cells and on S-Fuse cells.

#### mAbs

Sotrovimab was provided by CHR Orleans.

#### Cell lines

293T cells (ATCC CRL-3216) and U2OS cells (ATCCa HTB-96) were grown in complete DMEM medium (10% FCS, 1% PS). U2OS stably expressing ACE2 and the GFPsplit system (GFP1-10 and GFP11; S-Fuse cells) were previously described.^38^ Blasticidin (10 mg/mL) and puromycin (1 mg/mL) were used to select for ACE2 and GFPsplit transgenes expression, respectively. 293T cells stably expressing the SARS-CoV-2 Spike protein of D614G, Delta, BA.2 and BA.5 were generated by lentiviral transduction and selection with puromycin (1 mg/mL). Absence of mycoplasma contamination was confirmed in all cell lines with the Mycoalert Mycoplasma Detection Kit (Lonza). All cell lines were cultured at 37°C and 5% CO2.

#### Antigenic peptides

Two pools, designated S1 and S2, of 315 (158 + 157) peptides derived from a peptide scan (15-mers with 11 amino acid overlap) through the Spike glycoprotein of SARS-CoV-2 Omicron BA.1 strain as well as a pool of 102 peptides derived from a peptide scan (15-mers with 11 amino acid overlap) through the Nucleoprotein (N, Swiss-Prot ID: P0DTC9) of SARS-CoV-2 Wuhan strain were purchased from JPT-Peptide-Technologies. As control, we used a pool of 23 pan-HLA-presented, 9–10 amino acid-long, peptide epitopes from human cytomegalovirus, Epstein-Barr virus, and influenza virus (CEF) from Cellular Technology Limited. Lyophilized peptides were solubilized with tissue culture-grade DMSO.

### Method details

#### Serum cytokine concentration

Cytokines concentrations in serum were measured using the Meso Scale Discovery technology (an electrochemiluminescence-based solid-phase multiplex assay). IL-1RA, IL-6, IL-8, IL-10, IL-15, IL-18, IFNγ, CCL2, CCL3 and CXCL10 were quantified with the U-PLEX Biomarker Group 1 kit. IL1β and IFNα2a were measured with two S-PLEX assays and an R-PLEX was used for BCA-1/CXCL13. All assays were performed by a single operator according to the manufacturer’s instructions. Analyses were performed on the MESO QuickPlex SQ120 instrument and analyzed using the DISCOVERY WORKBENCH 4.0 software. Concentrations were interpolated from a four-parameter logistic standard curve.

#### PBMCs cryopreservation

PBMCs were obtained from EDTA tubes within 4 h after the venipuncture. Isolation was performed by density-gradient sedimentation using Ficoll-Paque (Pancoll, Pan-Biotech). PBMCs were frozen in heat-inactivated fetal bovine serum (Gibco FBS, ThermoFisher) containing 10% tissue culture-grade DMSO (Sigma). Cryovials containing 10-15.10^6^ cells were transferred at −80°C at a cooling rate of 0.2°C–1 °C/min using a cryopreservation module and transferred to liquid nitrogen after 24 to 96 h until use.

#### IFNγ enzyme-linked immunospot (ELISPOT) assay

PBMCs were thawed and allowed to rest for 20 ± 4 h in complete RPMI (RPMI 1640 with 10% heat-inactivated FBS, L-glutamine, 1 mM sodium pyruvate and PenStrep). Cells were counted on a LUNA-FL cell counter (Logos Biosystems) using acridine orange and propidium iodide dyes. ELISPOT assays were performed using the human IFNγ ELISPOT kit (Diaclone) according to the manufacturer’s protocol. For S1, S2 and N peptide pools, cells were seeded at a concentration of 4 × 10^5^/well and peptides were used at 0.125 μg/mL. The CEF pool was used at 0.25 μg/mL with a density of 2 × 10^5^ PBMC/well. As negative and positive controls, 4 × 10^5^ and 10^4^ cells/well were incubated with 0.125% DMSO and 0.5 μg/mL of anti-CD3/CD28 mAbs (clone HIT3a and CD28.2; BD Biosciences), respectively. Spots were enumerated using an automated spot counter (ImmunoSpot CTL S6 Ultra-V Analyzer; Cellular Technology Limited). Specific responses were calculated after averaging duplicate wells and subtracting non-specific responses (without peptides). The results were normalized with respect to the number of cells per well and expressed as IFNγ spot-forming cells (SFC) per million of PBMC. We excluded subject PBMC samples lacking response to all stimuli or having less than 75% of viable cells.

#### Neutralization

Neutralizing titers of authentic SARS-CoV-2 isolates were measured using the S-Fuse assay as previously described.^38^ All serum/plasma were heat-inactivated for 30 min at 56°C. Effective dose 50% (refer to as “titers”), in dilution, were calculated with a reconstructed curve using the percentage of the neutralization at the different concentrations. Neutralization was performed using a D614G strain as control and the corresponding infecting variant (Delta, BA.1, BA.2 or BA.5).

#### Antibody-dependent cellular cytotoxicity (ADCC)

ADCC was quantified using the ADCC Reporter Bioassay (Promega) as previously described.^17^ Briefly, 293T cells stably expressing the indicated Spikes (D614G, Delta, BA.1, BA.2 or BA.5) incubated overnight were co-cultured with Jurkat-CD16-NFAT-rLuc cells in presence or absence of sera/plasma at a final dilution of 1:30. Each sample was tested against D614G, the corresponding infecting variant and WT cells. We previously reported correlations between the ADCC Reporter Bioassay titers and an ADCC assay based on primary NK cells and cells infected with an authentic virus.^39^

#### qPCR and sequencing

The TaqPath COVID-19 RT-PCR assay (ThermoFisher, Waltham, USA) was used to detect viral target genes (ORF1ab, N, and S). Whole genome sequencing was performed using the xGen ARTIC nCoV-2019 Amplicon Panel v4.1 according to the Eco PCR tiling of SARS-CoV-2 virus protocol on the GridION device (Oxford Nanopore Technologies). Sequencing data were both base-called and demultiplexed using MinKNOW (Oxford Nanopore Technologies). Consensus sequences were generated using the ARTIC SARS-CoV-2 Workflow (v0.3.12) from EPI2ME labs. Briefly, reads are mapped to the Wuhan-Hu-1 reference genome (MN908947.3) using Minimap2. Alignment files are used to generate a consensus sequence, which is then polished using Medaka. Consensus sequences were annotated using Nextclade and strain assignment was performed using Pangolin. Resistance mutation emergence analyses were performed on the S gene using the PEPPER-Margin-DeepVariant pipeline (v0.8) to screen for single nucleotide variations (SNVs).

### Quantification and statistical analysis

#### Univariate and bivariate analysis

The descriptive data were presented as median with 25^th^ and 75^th^ percentile and percentages. Baseline characteristics were compared using the Mann-Whitney or Kruskal-Wallis tests for quantitative variables and Chi square or Fisher’s exact test for qualitative variables. No adjustment for multiple comparisons was applied in the bivariate analyses, as potential associations were subsequently evaluated in multivariable models accounting for confounding effects. A mixed-model with Tukey’s multiple comparisons post-hoc test were used for comparisons of quantitative variables between day 0 and day 7 and across treatment groups.

#### Multivariate analyses

Continuous variables were initially assessed for skewness and linearity with respect to the outcome using graphical methods (scatterplots, residual plots). Variables with skewed distributions were log_10_-transformed. Age was included as per 5 years variable for interpretation. To identify factors associated with SARS-CoV-2 N gene viral load at Day 7, a multivariable linear regression model was fitted. To identify factors associated with the emergence of spike gene mutations, a multivariable logistic regression model was used. Candidate covariates were first evaluated in bivariate analyses; variables with a *p*-value less than 0.10, as well as clinically relevant variables, were considered for inclusion in multivariable models. For the linear regression model, the 10 observations per variable rule was applied: with *n* = 107, the maximum number of predictors was limited to 11. To reduce the risk of overfitting given the limited number of mutation events, a penalized logistic regression (LASSO) with 5-fold cross-validation was performed as a sensitivity analysis. The results were compared with those from the conventional logistic regression model. Because treatment assignment was not randomized, potential confounding was addressed first by adjusting for key covariates in multivariable models and secondly by conducting a sensitivity analysis. Due to very small sample sizes, patients treated with Tixagevimab/Cilgavimab or Remdesivir were described descriptively only. Adjusted models were restricted to patients treated with Casirivimab/Imdevimab, Sotrovimab, or Nirmatrelvir/Ritonavir. For each multivariable analyses, we applied a 0.05 type I error rate.

#### Missing data

Missing covariate values were investigated for patterns and assumed to be missing at random (MAR). The multiple imputation by chained equations (MICE) procedure was used to impute missing data producing 50 imputations. Results were pooled by using Rubin’s rules. Variables with high missingness (>20%) were excluded for the main multivariable models.

#### Power calculations

Although formal power calculations were performed for the main clinical outcomes of the study,^40^ which determined the overall cohort size, they were not conducted for this immunological ancillary study, which was based on patient consent.

#### Software

All statistical analysis were performed using R version 4.4.1 and GraphPad Prism v10.

#### Additional resources

The COCOPREV (“Prévention des complications de la COVID-19”) cohort is registered on clinicaltrial.gov under the number: NCT04885452.
