# Supplementary material for: Viral clearance and escape during therapy of COVID-19 outpatients: A prospective cohort study
Source: iScience. 2025 Nov 26;28(12):114226. doi: 10.1016/j.isci.2025.114226 (PMC12767176; doi:10.1016/j.isci.2025.114226)
Supplement: Document S1. Figures S1 and S2 and Tables S1–S7 [file mmc1.pdf]

iScience, Volume *28*

Supplemental information

Viral clearance and escape during therapy of COVID-19 outpatients: A prospective cohort study

Guillaume Martin-Blondel, Paul Burgat, Valentin Leducq, Françoise Porrot, Andrea Cottignies-Calamarte, Alejandro De Cruz, Céline Dorival, Raphaelle Romieu-Mourez, Camille Chaubet, Vincent Cazaentre, Xavier Boumaza, Quentin Richier, Benjamin Gaborit, Francois Coustilleres, Vincent Dubée, Florence Ader, Youri Yordanov, Olivier Schwartz, Anne-Geneviève Marcelin, Clovis Lusivika-Nzinga, Fabrice Carrat, Cathia Soulié, Roland Liblau, Timothée Bruel, and for the ANRS 0003S CoCoPrev Study Group

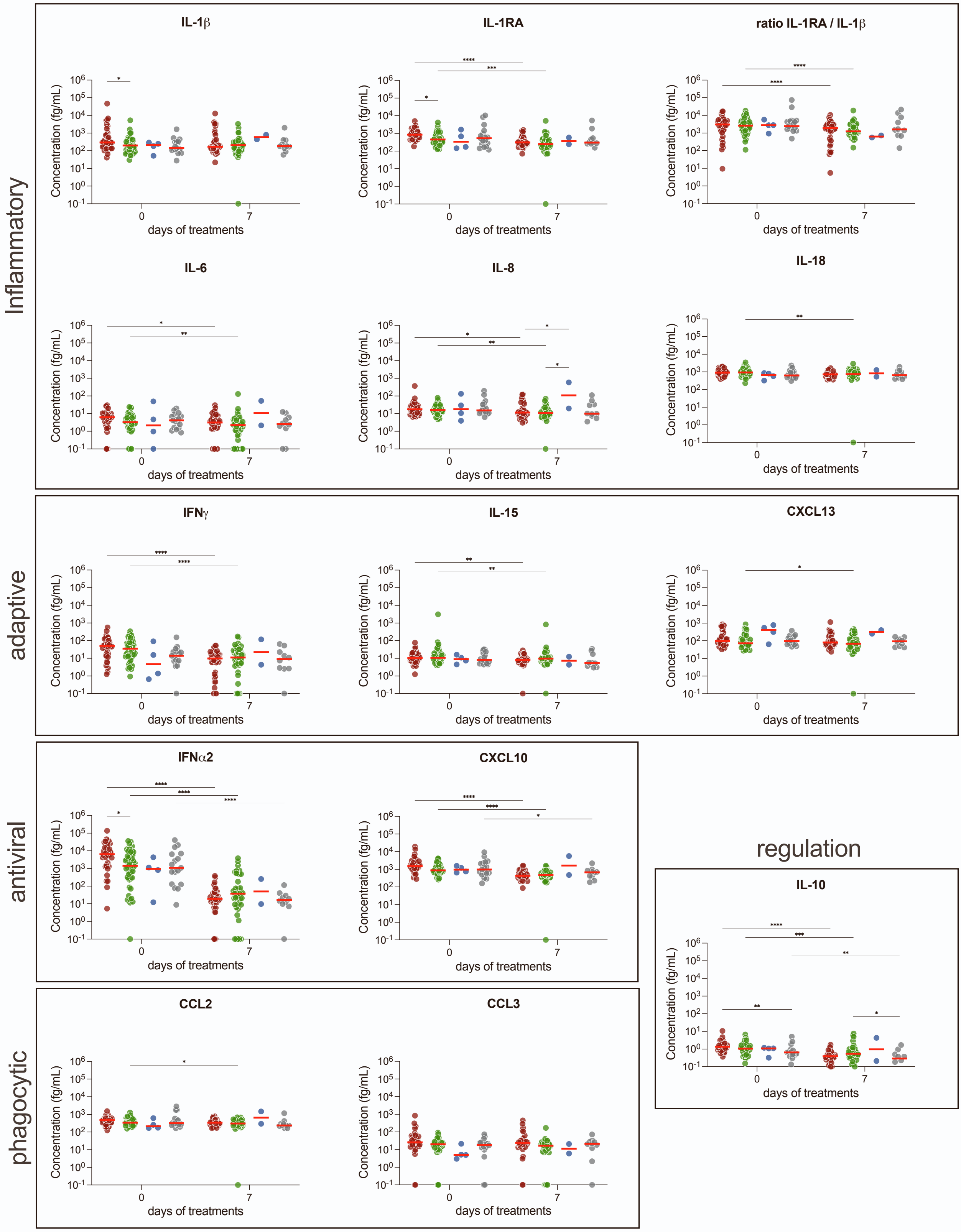

Supplementary Figure 1: Cytokine and chemokine levels in blood.

Cytokine concentrations are organized based on analysis of the inflammatory, adaptive, antiviral, phagocytic and regulative components of the immune response. Mixed-model with Tukey's multiple comparisons post-hoc test ; * $p<0.05$; ** $p<0.01$; *** $p<0.001$; **** $p<0.0001$.. Red bars indicate medians. The "Antivirals" group refers to Nirmatrelvir/ritonavir and Remdesivir-treated patients.

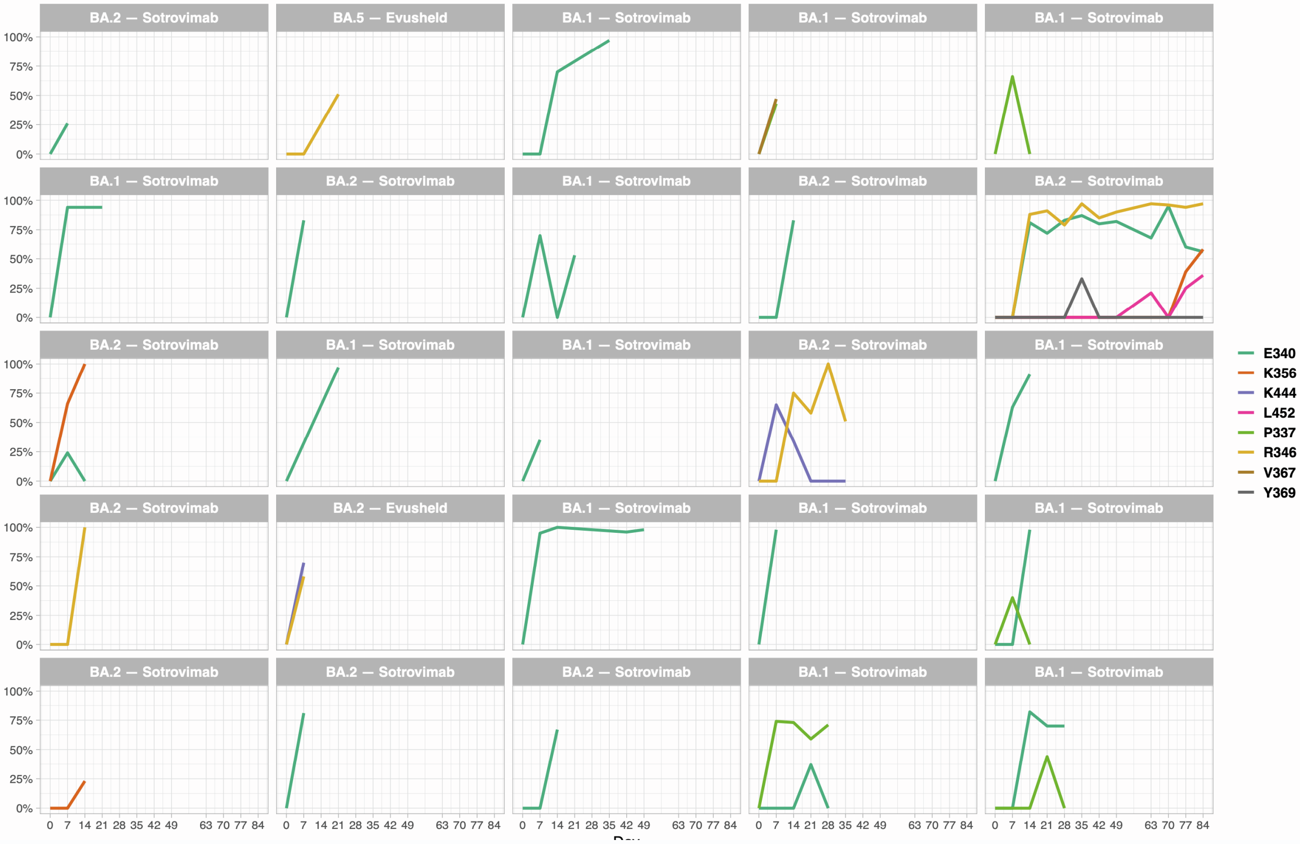

Supplementary Figure 2: Longitudinal dynamics of single nucleotide variations in patients with viral emergence.

Data are presented for 25 patients who developed mutations in the RBD domain of the spike protein, stratified by infecting variant and treatment received.

Supplementary Table 1: Baseline characteristics and outcome of the 107 consecutive patients included based on the emergence of mutations in the Spike gene during the follow-up without the 3 Remdesivir and the 4 Tixagevimab/Cilgavimab treated patients (N=107).

	All patients (n=107)	Emergence (n=23)	No emergence (n=84)	P value
**Median age (Q1-Q3)**	54.00 (44.00 - 69.00)	64.00 (52.00 - 70.00)	53.50 (42.00 - 67.50)	0.118
≥ 80 years old – N(%)	9 (100.0)	2 (22.2)	7 (77.8)	1.000
**Median BMI (Q1-Q3)**	26.00 (23.00 - 32.00)	24.00 (22.00 - 29.00)	28.00 (23.00 - 32.00)	0.189
Missing	9	0	9	
**Male gender (%)**	52 (100.0)	13 (25.0)	39 (75.0)	0.482
**Vaccination status (%)**				0.442
Complete (≥ 3 doses)	72 (100.0)	17 (23.6)	55 (76.4)	
Incomplete (≤ 2 doses) or unvaccinated	32 (100.0)	5 (15.6)	27 (84.4)	
Missing	3	1	2	
**Risk factor for severe COVID-19**				
**Immunocompromised patients (%)**	83 (100.0)	23 (27.7)	60 (72.3)	**0.002**
Ongoing chemotherapy	16 (100.0)	6 (37.5)	10 (62.5)	0.106
Solid organ transplantation	19 (100.0)	6 (31.6)	13 (68.4)	0.235
Bone marrow transplantation	5 (100.0)	2 (40.0)	3 (60.0)	0.292
Systemic lupus or vasculitis with immunosuppressive medications	5 (100.0)	1 (20.0)	4 (80.0)	1.000
Corticosteroids	9 (100.0)	3 (33.3)	6 (66.7)	0.401
Immunosuppressive therapy, including RTX	52 (100.0)	12 (23.1)	40 (76.9)	0.815
Cancer	7 (100.0)	2 (28.6)	5 (71.4)	0.641
**Comorbidities (%)**	62 (100.0)	12 (19.4)	50 (80.6)	0.635
Obesity BMI>30	26 (100.0)	4 (15.4)	22 (84.6)	0.584
COPD and chronic respiratory failure	4 (100.0)	2 (50.0)	2 (50.0)	0.201
High blood pressure	9 (100.0)	2 (22.2)	7 (77.8)	1.000
Congestive heart failure	6 (100.0)	2 (33.3)	4 (66.7)	0.607
Diabetes (type 1 and type 2)	11 (100.0)	1 (9.1)	10 (90.9)	0.450
Chronic kidney disease	13 (100.0)	2 (15.4)	11 (84.6)	0.730
Other chronic pathologies	17 (100.0)	4 (23.5)	13 (76.5)	0.758
**SARS-CoV-2 variant**				**<0.001**
Delta	35 (100.0)	0 (0.0)	35 (100.0)	
Omicron	71 (100.0)	23 (32.4)	48 (67.6)	
Omicron BA.1	28 (100.0)	13 (46.4)	15 (53.6)	
Omicron BA.2	38 (100.0)	10 (26.3)	28 (73.7)	
Other	5 (100.0)	0 (0.0)	5 (100.0)	
Missing	1	0	1	
**Severity of COVID-19 (%)**				0.633
Mild	99 (100.0)	20 (20.2)	79 (79.8)	
Moderate	7 (100.0)	2 (28.6)	5 (71.4)	
Missing	1	1	0	
**Median time between symptoms onset and initiation of treatment (days, Q1- Q3)**	3.00 (2.00 - 4.00)	3.00 (2.00 - 4.00)	3.00 (2.00 - 4.00)	0.867
Missing	16	1	15	
**Antiviral treatment received at D0 (%)**				**<0.001**
Sotrovimab	56 (100.0)	23 (41.1)	33 (58.9)	
Casirivimab/Imdevimab	37 (100.0)	0 (0.0)	37 (100.0)	
Nirmatrelvir/ritonavir	14 (100.0)	0 (0.0)	14 (100.0)	
**Median SARS-CoV-2 N gene viral load at D0 (log10 copies/ml, Q1-Q3)**	8.20 (7.58 - 9.02)	8.51 (7.97 - 9.13)	8.05 (7.19 - 8.84)	**0.022**
**Anti-N Ig G at D0**				1.000
Borderline (%)	2 (100.0)	0 (0.0)	2 (100.0)	
Positive (%)	3 (100.0)	1 (33.3)	2 (66.7)	
Negative (%)	90 (100.0)	22 (24.4)	68 (75.6)	
Median titer of anti-N Ig G (Q1-Q3)	0.99 (0.77 – 6.35)	6.35 (6.35 - 6.35)	0.88 (0.76 – 2.72)	0.800
Missing	12	0	12	
**Anti-S Ig G at D0**				0.089
Positive (%)	60 (100.0)	11 (18.3)	49 (81.7)	
Negative (%)	35 (100.0)	12 (34.3)	23 (65.7)	
Median titer of anti-S Ig G (BAU/mL, Q1-Q3)	33.90 (0.00 - 403.71)	4.08 (0.00 - 33.90)	95.64 (1.21 - 462.18)	**0.039**
Missing	12	0	12	
**Median serum concentrations at D0 (Q1-Q3)**				
IL-1b (fg/ml)	237.28 (112.48 - 402.00)	241.54 (126.97 - 468.39)	237.28 (105.13 - 391.01)	0.406
IL-1RA (pg/ml)	568.35 (347.28 - 1162.42)	557.75 (368.08 - 1153.03)	568.35 (344.96 - 1177.83)	0.697
IL-1RA/IL-1b ratio of fg/mL	2757.41 (1410.56 - 4708.07)	2513.86 (1410.56 - 3452.72)	2909.13 (1445.79 - 4736.47)	0.585
IL-6 (pg/ml)	3.96 (1.72 - 7.42)	3.39 (1.76 - 7.42)	4.21 (1.68 - 7.72)	0.601

IL-8 (pg/ml)	16.47 (10.97 - 27.08)	19.26 (13.55 - 25.86)	16.18 (10.87 - 28.25)	0.829
IL-15 (pg/ml)	10.63 (6.61 - 18.05)	10.75 (8.32 - 15.10)	10.59 (6.55 - 18.91)	0.710
IL-18 (pg/ml)	903.05 (676.06 - 1277.98)	1064.26 (796.19 - 1472.05)	896.22 (657.12 - 1223.39)	0.191
IL-10 (pg/ml)	1.08 (0.66 - 1.69)	0.97 (0.68 - 1.58)	1.09 (0.65 - 1.80)	0.989
IFN-g (pg/ml)	34.51 (12.50 - 64.42)	33.04 (16.17 - 55.89)	34.51 (9.86 - 69.67)	0.808
IFN-a 2a (pg/ml)	2730.02 (470.06 - 8660.88)	3705.18 (1240.10 - 12332.59)	2297.34 (301.55 - 7995.72)	0.261
CCL2 (pg/ml)	377.03 (275.75 - 538.63)	424.55 (309.34 - 543.33)	368.67 (262.35 - 530.18)	0.338
CCL3 (pg/ml)	20.73 (14.30 - 31.80)	20.37 (14.28 - 24.10)	21.17 (14.52 - 33.45)	0.530
CXCL10 (pg/ml)	1131.69 (698.96 - 1915.12)	1138.48 (746.52 - 1795.77)	1131.69 (666.09 - 1932.35)	0.815
CXCL13 (pg/ml)	80.96 (59.15 - 139.68)	88.31 (57.68 - 137.67)	79.22 (59.92 - 139.73)	0.591
Missing	9	5	4	
**Median serum concentrations at D7 (Q1-Q3)**				
IL-1b (fg/ml)	181.97 (111.36 - 342.18)	179.12 (89.21 - 307.64)	182.61 (112.74 - 355.21)	0.489
IL-1RA (pg/ml)	289.73 (190.68 - 439.86)	267.54 (164.24 - 431.43)	297.39 (206.43 - 483.65)	0.317
IL-1RA/IL-1b ratio of fg/mL	1605.55 (777.27 - 3029.25)	1241.53 (901.52 - 2806.27)	1638.75 (682.34 - 3098.07)	0.666
IL-6 (pg/ml)	2.38 (0.64 - 4.66)	1.38 (0.32 - 5.18)	2.47 (1.11 - 4.53)	0.381
IL-8 (pg/ml)	10.74 (6.45 - 17.55)	9.57 (6.20 - 17.94)	10.92 (6.58 - 17.40)	0.727
IL-15 (pg/ml)	8.35 (5.66 - 13.32)	9.95 (6.38 - 11.91)	8.26 (5.55 - 15.95)	0.781
IL-18 (pg/ml)	736.79 (562.35 - 1042.74)	861.48 (563.35 - 1195.18)	729.04 (560.59 - 988.15)	0.404
IL-10 (pg/ml)	0.46 (0.29 - 0.71)	0.47 (0.33 - 0.62)	0.46 (0.29 - 0.81)	1.000
IFN-g (pg/ml)	10.41 (2.85 - 23.49)	8.89 (5.37 - 34.42)	10.59 (2.06 - 23.28)	0.558
IFN-a 2a (pg/ml)	22.33 (9.50 - 67.03)	178.84 (36.75 - 634.92)	19.15 (8.95 - 41.74)	**0.002**
CCL2 (pg/ml)	313.99 (237.63 - 395.22)	317.19 (246.81 - 506.08)	310.65 (236.19 - 384.93)	0.397
CCL3 (pg/ml)	20.89 (10.35 - 28.48)	12.15 (8.49 - 20.42)	21.81 (13.57 - 30.31)	**0.028**
CXCL10 (pg/ml)	461.42 (339.71 - 702.69)	490.33 (388.24 - 760.88)	433.59 (334.41 - 690.39)	0.300
CXCL13 (pg/ml)	72.40 (49.09 - 166.55)	60.68 (25.52 - 117.50)	73.72 (52.72 - 170.28)	0.230
Missing	24	8	16	
**Median T cell specific response at D0 (Q1-Q3)**				
S1-specific T cells / 10^6 PBMC	22.00 (0.00 - 135.00)	12.00 (0.00 - 197.00)	23.50 (0.00 - 135.00)	0.675
Missing	18	4	14	
S2-specific T cells / 10^6 PBMC	15.00 (0.00 - 137.00)	20.00 (0.00 - 102.00)	15.00 (0.00 - 160.00)	0.556
Missing	21	4	17	
N-specific T cells / 10^6 PBMC	0.00 (0.00 - 10.00)	0.00 (0.00 - 2.00)	0.00 (0.00 - 10.00)	0.239
Missing	28	5	23	
**Median T cell specific response at D7 (Q1-Q3)**				
S1-specific T cells / 10^6 PBMC	85.00 (15.00 - 337.00)	85.00 (30.00 - 337.00)	86.00 (15.00 - 305.00)	0.944
Missing	16	2	14	
S2-specific T cells / 10^6 PBMC	103.50 (23.50 - 389.50)	115.00 (50.00 - 453.50)	103.50 (20.00 - 389.50)	0.697
Missing	19	3	16	
N-specific T cells / 10^6 PBMC	25.00 (0.00 - 80.00)	5.00 (0.00 - 75.00)	25.00 (5.00 - 80.00)	0.279
Missing	19	2	17	
**Serum median neutralization titer (Q1-Q3)**				
D0 on D614G	15.00 (15.00 - 941.40)	15.00 (15.00 - 46.52)	75.03 (15.00 - 1096.50)	0.060
D0 on the infecting variant	15.00 (15.00 - 94.99)	15.00 (15.00 - 15.00)	15.00 (15.00 - 111.10)	**0.024**
D7 on D614G	9083.00 (718.20 - 187777.00)	531.70 (367.00 - 915.20)	38549.50 (1322.50 - 226711.50)	**<0.001**
D7 on the infecting variant	839.60 (56.22 - 133432.00)	48.21 (15.00 - 116.90)	4432.00 (135.50 - 179926.50)	**<0.001**
**Serum median ADCC titer (Q1-Q3)**				
D0 on D614G	1.22 (1.12 - 1.68)	1.12 (1.10 - 1.32)	1.30 (1.15 - 1.76)	**0.016**
D0 on the infecting variant	1.28 (1.13 - 1.74)	1.16 (1.12 - 1.29)	1.36 (1.15 - 1.79)	**0.042**
D7 on D614G	5.25 (3.60 - 7.98)	8.67 (6.10 - 9.87)	4.93 (3.56 - 6.92)	**<0.001**
D7 on the infecting variant	4.83 (2.97 - 7.10)	7.30 (4.56 - 10.97)	4.12 (2.70 - 6.72)	**<0.001**
**SARS-CoV-2 N gene viral load at D7 (log10 copies/ml, Q1-Q3)**	4.88 (3.45 - 6.78)	7.74 (6.95 - 8.03)	4.10 (2.91 - 5.91)	**<0.001**
Missing	2	0	2	
**Day 7 outcome (% of patients with available data)**	104/107 (97.2)	22/23 (95.7)	82/84 (97.6)	0.170
Worsening of symptoms (%)	1 (100.0)	1 (100.0)	0 (0.0)	
No Evolution of symptoms (%)	15 (100.0)	4 (26.7)	11 (73.3)	
Improvement of symptoms (%)	88 (100.0)	17 (19.3)	71 (80.7)	
COVID-19–related hospitalization (%)	1 (100.0)	0 (0.0)	1 (100.0)	1.000
**Day 28 outcome (% of patients with available data)**	101/107 (94.4)	21/23 (91.3)	80/84 (95.2)	0.201
Worsening of symptoms (%)	3 (100.0)	1 (33.3)	2 (66.7)	
No Evolution of symptoms (%)	4 (100.0)	2 (50.0)	2 (50.0)	
Improvement of symptoms (%)	94 (100.0)	18 (19.1)	76 (80.9)	
COVID-19–related hospitalization (%)	1 (100.0)	0 (0.0)	1 (100.0)	1.000

Supplementary Table 2: Linear regression analysis of factors associated with SARS-CoV-2 N gene viral load at day 7 (log10 cp/ml) without the 3 Remdesivir and the 4 Tixagevimab/Cilgavimab treated patients (N=107) with 14 variables.

	Bivariate analysis<br>Coefficient [95%CI] (P-value)	Multivariable analysis<br>Coefficient [95%CI] (P-value)
Sotrovimab	Ref	Ref
Casirivimab/Imdevimab	**-2.04 [-3.02 ; -1.07] (<0.001)**	0.45 [-1.80 ; 2.69] (0.693)
**Nirmatrelvir/ritonavir**	**-3.27 [-4.65 ; -1.90] (<0.001)**	**-3.11 [-4.68 ; -1.54] (<0.001)**
Age (per 5 years)	**0.18 [0.02 ; 0.33] (0.025)**	0.09 [-0.05 ; 0.24] (0.202)
BMI (kg/m²)	**-0.09 [-0.16 ; -0.02] (0.008)**	-0.05 [-0.12 ; 0.01] (0.111)
Gender (male vs female)	**1.20 [0.21 ; 2.18] (0.018)**	0.50 [-0.38 ; 1.38] (0.259)
Immunocompromised patients (vs non immunocompromised)	**1.61 [0.44 ; 2.77] (0.008)**	0.39 [-0.77 ; 1.55] (0.502)
Patients with comorbidities (vs no comorbidities)	**-0.88 [-1.90 ; 0.15] (0.094)**	-0.20 [-1.13 ; 0.73] (0.674)
Vaccine status incomplete (vs complete)	-0.21 [-1.31 ; 0.88] (0.699)	0.06 [-0.94 ; 1.07] (0.900)
Serum IFN-α2a concentration at D0 (log10 pg/ml)	0.07 [-0.42 ; 0.56] (0.773)	-0.16 [-0.64 ; 0.33] (0.528)
Serum CXCL13 concentration at D0 (log10 pg/ml)	**1.44 [0.07 ; 2.82] (0.040)**	0.86 [-0.43 ; 2.07] (0.197)
Titer of anti-S Ig G at D0 (log10 BAU/mL)	**-0.44 [-0.83 ; -0.05] (0.029)**	0.19 [-0.27 ; 0.65] (0.415)
Serum neutralization of the infecting variant at D0 (log10 titer)	**-1.23 [-1.86 ; -0.59] (<0.001)**	-0.43 [-1.19 ; 0.33] (0.262)
**SARS-CoV-2 N gene viral load at D0 (log10 cp/ml)**	**0.75 [0.32 ; 1.17] (0.001)**	**0.60 [0.21 ; 0.99] (0.003)**
**Serum neutralization of the infecting variant at D7 (log10 titer)**	**-0.62 [-0.91 ; -0.34] (<0.001)**	**-0.72 [-1.34 ; -0.10] (0.024)**
Serum ADCC of the infecting variant at D7 (fold-change)	**0.21 [0.05 ; 0.36] (0.009)**	-0.06 [-0.21 ; 0.10] (0.466)

Supplementary Table 3: Linear regression analysis of factors associated with SARS-CoV-2 N gene viral load at day 7 (log10 cp/ml) in the immunocompromised patients without the 3 Remdesivir and the 4 Tixagevimab/Cilgavimab treated patients (N=83).

	Bivariate analysis<br>Coefficient [95%CI] (P-value)	Multivariable analysis<br>Coefficient [95%CI] (P-value)
Sotrovimab	Ref	Ref
Casirivimab/Imdevimab	**-1.94 [-3.08 ; -0.80] (0.001)**	-0.03 [-2.42 ; 2.36] (0.980)
**Nirmatrelvir/ritonavir**	**-3.65 [-5.68 ; -1.61] (0.001)**	**-3.26 [-5.09 ; -1.43] (0.001)**
**Age (per 5 years)**	**0.25 [0.06 ; 0.44] (0.011)**	**0.20 [0.03 ; 0.36] (0.022)**
**BMI (kg/m²)**	**-0.12 [-0.21 ; -0.03] (0.012)**	**-0.10 [-0.17 ; -0.02] (0.013)**
Gender (male vs female)	**1.21 [0.09 ; 2.33] (0.034)**	0.26 [-0.69 ; 1.22] (0.586)
Vaccine status incomplete (vs complete)	-0.16 [-1.45 ; 1.12] (0.802)	-0.48 [-1.52 ; 0.55] (0.355)
**Serum CXCL13 concentration at D0 (log10 pg/ml)**	**1.50 [0.01 ; 2.99] (0.049)**	**1.23 [0.02 ; 2.44] (0.046)**
Serum neutralization of the infecting variant at D0 (log10 titer)	**-1.35 [-2.19 ; -0.51] (0.002)**	-0.45 [-1.31 ; 0.40] (0.295)
**SARS-CoV-2 N gene viral load at D0 (log10 cp/ml)**	**0.74 [0.29 ; 1.19] (0.002)**	**0.52 [0.13 ; 0.92] (0.010)**
**Serum neutralization of the infecting variant at D7 (log10 titer)**	**-0.66 [-0.99 ; -0.34] (<0.001)**	**-0.71 [-1.40 ; -0.02] (0.045)**
Serum ADCC of the infecting variant at D7 (fold-change)	**0.21 [0.03 ; 0.39] (0.021)**	-0.12 [-0.30 ; 0.06] (0.184)

Supplementary Table 4: Multivariable linear regression of factor associated with SARS-CoV-2 N gene viral load at day 7 (log10 cp/ml) for patients with complete vaccine status (N=76).

	**Multivariable analysis Coefficient [95%CI] (P-value)**
Sotrovimab	Ref
Casirivimab/Imdevimab	-1.76 [-4.4 ; 0.88] (0.187)
**Nirmatrelvir/ritonavir**	**-3.52 [-5.06 ; -1.98] (<0.001)**
Age (per 5 years)	0.11 [-0.06 ; 0.28] (0.210)
**BMI (kg/m²)**	**-0.08 [-0.15 ; 0.00] (0.049)**
Gender (male vs female)	0.36 [-0.61 ; 1.32] (0.465)
Immunocompromised patients (vs non immunocompromised)	1.38 [0.02 ; 2.75] (0.047)
Serum CXCL13 concentration at D0 (log10 pg/ml)	0.97 [-0.37 ; 2.31] (0.152)
Serum neutralization of the infecting variant at D0 (log10 titer)	0.09 [-0.84 ; 1.02] (0.848)
**SARS-CoV-2 N gene viral load at D0 (log10 cp/ml)**	**0.93 [0.44 ; 1.41] (<0.001)**
Serum neutralization of the infecting variant at D7 (log10 titer)	-0.24 [-0.98 ; 0.50] (0.515)
Serum ADCC of the infecting variant at D7 (fold-change)	-0.05 [-0.22 ; 0.13] (0.604)

Supplementary Table 5: Multivariable linear regression of factor associated with SARS-CoV-2 N gene viral load at day 7 (log10 cp/ml) for patients younger than 80 years (N=98).

	**Multivariable analysis Coefficient [95%CI] (P-value)**
Sotrovimab	Ref
Casirivimab/Imdevimab	0.38 [-1.89 ; 2.65] (0.742)
**Nirmatrelvir/ritonavir**	**-2.78 [-4.40 ; -1.16] (0.001)**
BMI (kg/m²)	-0.06 [-0.12 ; 0.00] (0.057)
Gender (male vs female)	0.67 [-0.19 ; 1.54] (0.127)
Immunocompromised patients (vs non immunocompromised)	-0..30 [-1.52 ; 0.92] (0.625)
Vaccine status incomplete (vs complete)	-0.06 [-1.08 ; 0.97] (0.914)
Serum CXCL13 concentration at D0 (log10 pg/ml)	1.16 [-0.08 ; 2.40] (0.066)
Serum neutralization of the infecting variant at D0 (log10 titer)	-0.39 [-1.06 ; 0.29] (0.258)
**SARS-CoV-2 N gene viral load at D0 (log10 cp/ml)**	**0.50 [0.10 ; 0.91] (0.016)**
**Serum neutralization of the infecting variant at D7 (log10 titer)**	**-0.84 [-1.49 ; -0.18] (0.013)**
Serum ADCC of the infecting variant at D7 (fold-change)	-0.12 [-0.29 ; 0.04] (0.143)

Supplementary Table 6: Logistic regression analysis of factors associated with emergence of amino acid-substitution mutation in immunocompromised patients without the 3 Remdesivir and the 4 Tixagevimab/Cilgavimab treated patients (N=83) only with the variables selected by the LASSO with imputing data (23 emergence vs 60 no emergence).

	**Bivariate analysis OR [95%CI] (P-value)**	**Multivariable analysis OR [95%CI] (P-value)**
**Serum neutralization of the infecting variant at D7 (log10 titer)**	**0.29 [0.13 – 0.51] (<0.001)**	**0.46 [0.20 – 0.85] (0.031)**
Serum ADCC of the infecting variant at D7 (fold-change)	**1.35 [1.15 – 1.63] (<0.001)**	1.24 [0.97 – 1.65] (0.108)
**SARS-CoV-2 N gene viral load at D7 (log10 cp/ml)**	**2.75 [1.79 – 4.90] (<0.001)**	**2.62 [1.59 – 5.19] (0.001)**

Supplementary Table 7: sequencing depth

Sample	Mean Depth in RBD	Mean Depth in Spike
ACYX_J0	13503	10016
ACYX_J3	16277	10262
ACYX_J5	10867	7359
ACYX_J7	25479	19866
AFOM_J0	1839	1912
AFOM_J3	1915	1973
AFOM_J5	2417	2678
AFOM_J7	886	952
AMUR_J0	1991	3153
AMUR_J7	1816	2813
ANUG_J0	635	1701
ANUG_J3	59	523
APUB_J0	4756	5834
APUB_J14	25	13
BIBI_J0	1065	2218
BIBI_J7	1167	2327
BOCI_J0	1302	2550
BOCI_J14	295	1011
BOCI_J21	108	478
BOCI_J7	1188	2073
CUVA_J0	2941	5401
CUVA_J21	168	256
CUVA_J7	3351	3336
EDAK_J0	307	660
EDAK_J7	137	587
EFIW_J00	1434	1237
EFIW_J07	191	176
EJIX_J0	314	582
EJIX_J14	175	335
EJIX_J35	97	206
EJIX_J3	283	800
EJIX_J7	226	519
EMEZ_J0	1214	2462
EMEZ_J7	164	515
ENAZ_J0	936	2006
ENAZ_J5	265	1251
ENAZ_J7	196	915
ERAQ_J0	189	1019
ESAH_J0	294	752
ESAH_J7	813	1900
FIHU_J0	10142	7585
FIHU_J14	2832	1203
FIHU_J7	5001	3336
GEJU_J0	6217	8185
GEJU_J7	939	2223

GUXY_J0	1957	2628
GUXY_J14	572	634
GUXY_J7	3684	3504
HAGY_J0	1189	1546
HAGY_J14	351	821
HAGY_J7	298	786
IDAP_J0	303	353
IDAP_J3	264	203
IFEL_J0	4291	4970
IFEL_J7	609	1027
IHAB_J07	1465	1137
IHAB_J0	4378	5513
ILUD_J0	2304	3880
ILUD_J14	1271	2008
ILUD_J7	1067	1615
IQOT_J0	2922	3541
IQOT_J21	307	977
IQOT_J7	438	1102
IRIT_J0	2140	3342
IRIT_J7	1700	3323
IROL_J0	4731	6354
IROL_J14	159	490
IROL_J21	202	882
IROL_J7	224	605
ITUF_J0	571	1426
ITUF_J14	181	479
ITUF_J7	697	1472
IVOK_J0	1212	2410
IVOK_J14	1510	3311
IVOK_J21	1291	2672
IVOK_J28	26	1361
IVOK_J35	163	1863
IVOK_J42	87	1596
IVOK_J49	105	1815
IVOK_J63	1889	1276
IVOK_J70	1986	1596
IVOK_J77	2478	1701
IVOK_J7	1259	2384
IVOK_J84	1672	1194
JEVO_J0	5202	5973
JOXO_J0	277	209
JOXO_J14	462	212
JOXO_J7	65	38
JYXU_J0	4509	5803
JYXU_J3	5018	4002
JYXU_J5	9653	6474
KIBO_J0	237	986
KIBO_J7	214	1147
KIPO_J0	1526	2722
KIPO_J7	1540	3664
LAPU_J0	283	808

LAPU_J14	256	837
LAPU_J7	472	950
NUVE_J0	2128	2656
NUVE_J21	355	1433
OKIR_J0	1320	1162
OKIR_J7	452	381
OQAQ_J0	2766	4143
OQAQ_J7	971	1989
OSAZ_J0	1418	783
OSAZ_J3	437	245
OSAZ_J5	143	72
OSEL_J0	320	1467
OSEL_J7	307	1336
OSUB_J0	565	1416
OSUB_J14	1082	1961
OSUB_J21	366	833
OSUB_J28	137	410
OSUB_J35	138	409
OSUB_J42	55	183
OSUB_J7	218	287
OWOQ_J0	622	1194
OWOQ_J14	292	459
OWOQ_J7	753	1535
PODY_J0	1640	2138
PODY_J7	719	1141
QAPU_J0	1625	3013
QAPU_J14	79	621
QAPU_J7	208	836
QOBY_J0	4604	5340
QOQY_J0	294	1931
QOQY_J14	269	1136
QOQY_J7	147	892
SISE_J0	2230	4340
SISE_J7	4079	4318
TAGE_J0	3263	4092
TAGE_J7	4350	4033
TALU_J0	634	1272
TALU_J14	344	1137
TALU_J7	805	1541
TEKO_J0	105	285
TEKO_J14	1131	2333
TEKO_J28	705	1091
TEKO_J42	364	682
TEKO_J49	261	594
TEKO_J7	344	773
TYPE_J0	2954	3717
TYPE_J7	388	1636
UDIV_J0	471	905
UDIV_J7	127	490
UFAZ_J0	896	1620
UFAZ_J3	49	185

UJIQ_J0	43	18
UJIQ_J7	1017	569
UMUW_J0	920	1649
UMUW_J14	98	672
UMUW_J7	863	1501
UTYM_J0	1218	2340
UTYM_J14	396	996
UTYM_J7	698	1263
UWEG_J0	479	973
UWEG_J7	295	329
UXAP_J0	1948	3147
UXAP_J7	870	1495
UZUL_J0	699	1268
UZUL_J7	2005	3185
VASO_J0	23	606
VASO_J7	63	798
VUTI_J0	1545	2920
VUTI_J14	1287	2458
VUTI_J7	1048	2185
WEFE_J0	559	331
WEFE_J21	164	80
WEFE_J7	2183	906
WYBE_J0	299	773
WYBE_J14	527	1204
WYBE_J21	165	330
WYBE_J28	29	38
WYBE_J7	723	1474
XANA_J07	136	107
XANA_J0	1778	1560
XEWA_J0	2085	2488
XEWA_J7	152	466
XOJA_J0	3158	4397
XOJA_J14	216	631
XOJA_J21	226	756
XOJA_J7	503	1764
YDAD_J0	4736	6638
YDAD_J7	49	195
YMUV_J0	3469	4349
YMUV_J7	2146	996
YMYB_J0	1220	1119
YMYB_J7	21	21
YQYH_J0	3113	3602
YQYH_J14	1184	1725
YQYH_J21	2097	2730
YQYH_J28	636	1223
YQYH_J7	1829	2391
